# Inhibitory effects of armepavine against hepatic fibrosis in rats

**DOI:** 10.1186/1423-0127-16-78

**Published:** 2009-09-02

**Authors:** Ting-Chun Weng, Chien-Chang Shen, Yung-Tsung Chiu, Yun-Lian Lin, Cheng-Deng Kuo, Yi-Tsau Huang

**Affiliations:** 1 Institute of Traditional Medicine, School of Medicine, National Yang-Ming University, Taipei, Taiwan, Republic of China; 2 National Research Institute of Chinese Medicine, Taipei, Taiwan, Republic of China; 3 Department of Medical Research and Education, Taichung Veterans General Hospital, Taichung, Taiwan, Republic of China; 4 Department of Medical Research and Education, Taipei Veterans General Hospital, Taipei, Taiwan, Republic of China

## Abstract

Activation of hepatic stellate cells (HSCs) plays a crucial role in liver fibrogenesis. armepavine (Arm, C_19_H_23_O_3_N), an active compound from *Nelumbo nucifera*, has been shown to exert immunosuppressive effects on T lymphocytes and on lupus nephritic mice. The aim of this study was to investigate whether Arm could exert anti-hepatic fibrogenic effects *in vitro *and *in vivo*. A cell line of rat HSCs (HSC-T6) was stimulated with tumor necrosis factor-α (TNF-α) or lipopolysaccharide (LPS) to evaluate the inhibitory effects of Arm. An *in vivo *therapeutic study was conducted in bile duct-ligated (BDL) rats. BDL rats were given Arm (3 or 10 mg/kg) by gavage twice daily for 3 weeks starting from the onset of BDL. Liver sections were taken for fibrosis scoring, immuno-fluorescence staining and quantitative real-time mRNA measurements. *In vitro*, Arm (1-10 μM) concentration-dependently attenuated TNF-α- and LPS-stimulated α-SMA protein expression and AP-1 activation by HSC-T6 cells without adverse cytotoxicity. Arm also suppressed TNF-α-induced collagen collagen deposition, NFκB activation and MAPK (p38, ERK1/2, and JNK) phosphorylations. *In vivo*, Arm treatment significantly reduced plasma AST and ALT levels, hepatic α-SMA expression and collagen contents, and fibrosis scores of BDL rats as compared with vehicle treatment. Moreover, Arm attenuated the mRNA expression levels of *col 1α2, TGF-β1*, *TIMP-1, ICAM-1*, *iNOS*, and *IL-6 *genes, but up-regulated *metallothionein *genes. Our study results showed that Arm exerted both *in vitro *and *in vivo *antifibrotic effects in rats, possibly through anti-NF-κB activation pathways.

## Background

Liver fibrosis is a wound-healing response to various chronic liver injuries, including alcoholism, persistent viral and helminthic infections, and hereditary metal overload [1,2]. Activation of hepatic stellate cells (HSCs) plays a crucial role in the development of liver fibrosis [1-5]. During the activation process, HSCs undergo phenotype transformation from vitamin-A-storing quiescent cells to myofibroblast-like activated cells [1-4]. Activated HSCs are proliferative and fibrogenic, with accumulation of extracellular matrix (ECM), including type I collagen. Prolonged liver injury and inflammation result in hepatocyte damage, which triggers activation of HSCs and recruitment of inflammatory cells such as macrophages into the liver by paracrine secretion of cytokines [1,2]. Furthermore, activated HSCs have been implicated in hepatic inflammation through their ability to secrete cytokines and chemokines, and express adhesion molecules [1-4].

NFκB is an essential regulator of the expression of a number of genes involved in immune, inflammatory, and growth responses [6-8]. In most cells under normal conditions, NFκB exists in a latent state in the cytosol and is bound to inhibitory proteins including IκBα that mask a nuclear localization signal. Cytokines such as TNF-α activate NFκB signaling via the activation of the IκB-kinase (IKK) complex and subsequently phosphorylate and thereby degrade the IκBα protein, releasing the cytosolic dimer p65-p50 to translocate into the nucleus to activate transcription of various genes including *inducible nitric oxide synthase *(*iNOS*) and *intercellular adhension molecule *(*ICAM*)-*1 *[6-8]. Several *in vitro *studies showed that DNA binding activity of NFκB is demonstrated in activated but not in quiescent HSCs, and activation of HSCs is associated with the nuclear translocation of NFκB [9,10]. These observations provide functional support for a critical role of NFκB in the activation of HSCs.

*Nelumbo nucifera *is a common edible and medicinal plant used in Asia for the treatment of diarrhea, bleeding, fever, and infection [11-13]. Extracts from *Nelumbo nucifera *have been shown to exert antioxidant or free radical scavenging activities [12,14,15]. Armepavine (Arm, C_19_H_23_O_3_N), an active compound from *Nelumbo nucifera*, has been shown to exert not only anti-inflammatory effects on human peripheral blood mononuclear cells [16,17], but also immunosuppressive effects on T lymphocytes and on lupus nephritic mice [13]. In addition, Arm has been reported to induce apoptosis in leukemia cells [18]. However, the potential of Arm as an agent against hepatic fibrosis and its mechanisms of action remain to be clarified. Recently, we have reported that fibrosis-related gene transcripts were induced in the liver of BDL rats [19,20], and the TNF-α related signaling pathway in HSCs could be a therapeutic target. [21,22]. In the present study, we investigated the *in vitro *effects of Arm on TNF-α-induced NFκB activation in HSCs and *in vivo *anti-fibrotic effects in BDL rats.

## Methods

### Armepavine (Arm) solution and chemicals

Arm was obtained from Dr. Chien-Chang Shen (NRICM, Taiwan, ROC), according to the preparation procedures published before [13,23]. Armepavine synthesized by our method was a racemate with a purity of >95% and was confirmed by NMR analysis. Its NMR spectra were identical with those of (*S*)-armepavine. This synthesis has been repeated several times and gave the same results. For *in vitro *experiments, Arm was dissolved in dimethyl sulfoxide (DMSO) and diluted with medium to give a DMSO concentration below 0.1%. For *in vivo *experiments, Arm was mixed with 0.7% carboxyl methyl cellulose (CMC). Silymarin and other chemicals were from Sigma Chemical Co (St. Louis, MO, USA). Silymarin was also mixed with 0.7% CMC for *in vivo *administration.

### HSC-T6 cell line

The HSC-T6 cell line, a generous gift of Prof. S.L. Friedman, is an immortalized rat HSCs which are transfected by the large T-antigen of SV40 vector containing a Rous sarcoma virus promoter [24]. Bioassays of HSC-T6 activation have been previously established and reported by our group [21,22]. HSC-T6 cells were maintained in Waymouth's medium (containing 10% FBS, pH 7.0) at 37°C in 5% CO_2_/95% air. 90% confluent monolayer of HSCs were passaged by trypinization and HSCs were plated in 75T culture flask at a number of 1 × 10^6 ^cells per flask in Waymouth's medium containing 10% FBS and incubated under 5% CO_2 _in air at 37°C.

### Evaluation of cytotoxicity of Arm in HSCs

The potential of cytotoxicity of Arm was assessed by the MTT assay. Briefly, HSCs were incubated in 24-well plates containing Waymouth's MB752/1 medium (FBS-free) with or without Arm at different concentrations for 24 hrs at 37°C, with addition of minimum essential medium containing 0.1 mg/ml MTT in the last hour. After discarding medium, the formazan particle was dissolved with DMSO. The extent of reduction of 3-(4,5-dimethylthiazol-2-yl)-2,5- diphenyltetrazolium bromide (MTT) to formazan within cells was quantitated by measuring optical density at 540 nm by using enzyme-linked immunosorbent assay reader [22,25].

### Luciferase assays in transiently transfected HSCs

10^5 ^cells/well were seeded on 24-well plates the day before transfection. Plasmid NFκB-Luc and AP-1-Luc (1 μg/well) (Strategene, La Jolla, CA) and pRL-SV40 (0.2 μg/well) (Promega, Madison. USA) were transfected into cells by Fugene 6 (Roche, Indianapolis, IN, USA). The pNFκB-Luc and the pAP-1-Luc consist of NFκB and AP-1 binding regions. Plasmid pRL-SV40 served as an internal control to normalize the transfection efficiency. After treatment with TNF-α, LPS or drugs for 24 hrs in 5% CO_2 _incubator at 37°C, cells were harvested and lysed in 100 μl of lysis reagent. 20 μl of cell lysate was then mixed with 100 μl of luciferin before luminescence detection. The intensity of luciferase activity was measured with AutoLumat LB953 (Berthold technologies, Bad Wildbad, Germany). The luciferase assay kits were purchased from Promega (Madison. USA) [21,22].

### Western blot analyses

In brief, cytoplasmic and nuclear extracts of HSCs were prepared by washing ice-cold PBS twice. Then 100-μl lysis buffer A (10-mM HEPES, 10-mM KCl, 0.1-mM EDTA, 1-mM dithiothreitol (DTT), 0.5-mM phenylmethanesulfonyl fluoride (PMSF) in distilled water) was added into each dish with incubation on ice for 10 min. Cell lysates were collected into 1.5-ml tubes and were centrifuged at 15,000 × *g*, 4°C, 20 min. The supernatant (protein of cytoplasm) and lysate (protein of nucleus) were collected and 50-μl lysis buffer B (20-mM HEPES, 0.4-M NaCl, 25% glycerol, 1-mM EDTA, 1-mM DTT, 0.5-mM PMSF in distilled water) was added into the lysate of nucleus protein. The cell suspension was votexed for 10 seconds and were allowed to gently agitate for 30 min at 4°C and then centrifuged at 15,000 × *g *for 10 min at 4°C. The supernatant, containing the nuclear extract fraction, was transferred to a fresh 1.5-ml tube for NFκB translocation assay. Fifty μg of proteins each of cytoplasmic fraction from buffer A and nuclear fraction from buffer B were separated on a 10% SDS-PAGE and transferred onto Immobilon-PVDF (Millipore, Bedford, MA, USA) in a transfer buffer (6.2 mM boric acid, pH 8.0). Blots were incubated initially with blocking buffer (5% BSA) for 1 hour at room temperature, and then with specific primary antibodies against mouse pIκBα, mouse α-SMA, mouse α-tubulin, mouse p65 and mouse PCNA (Santa Cruz Biotechnology, Santa Cruz, California, CA, USA); and mitogen-activated protein kinase (MAPK) phosphorylation levels were determined by specific primary antibodies against p38, extracellular signal-regulated kinase (ERK)1/2 and c-jun N-terminal kinas (JNK) 1/2 (Cell Signaling Inc, USA). β-Actin was used as an internal control for equal protein loading. The protocols of our previous studies [26] were followed to measure phosphorylation of ERK, p38 MAPK, and JNK.

Primary antibodies had been diluted (1:10000) with Tris-buffered saline-Tween 20 (TBS-T) containing 1% non-fat milk. After primary antibody incubation, the blots were washed with TBS-T for 1 hr and incubated with specific second antibody conjugated with horseradish peroxidase (Becton Dickinson, Franklin Lakes, NJ, USA) for 1 hr at room temperature. After the washing of the secondary antibodies (1:2000) with TBS-T, immunodetection was performed, using an enhanced chemiluminescence kit for Western blot detection (Amersham Pharmacia Biotech, Buckinghamshire, U.K.). Film exposure ranged from a few seconds to 5 min.

### Quantification of collagen deposition by cultured HSCs

HSCs (in serum-free medium) were co-treated with TNF-α (10 ng/ml) and armepavine for 24 hrs. Cells were washed and collagen deposited in the wells was assayed using the Sircol collagen assay kit (Biocolor, Belfast, Nothern Ireland) according to the manufacturer's instructions [21]. The Sircol dye-collagen complex was dissolved in 0.5% sodium hydroxide after wash twice with ethanol. Collagen was quantitated by spectrophotometry at 540 nm and results were expressed as percentage of the untreated controls.

### Hepato-fibrotic rats

Hepatic fibrosis was induced by bile duct ligation (BDL) in male Sprague-Dawley rats (250~300 g) and we have recently documented changes in molecular and cell biological parameters related to fibrosis in these rats [19,20]. A double ligation of the bile duct was performed in rats under anesthesia by a proximal ligature around the bile duct in the hilus of the liver and by a distal ligature close to its entry into the duodenum. A cut was then made between ligatures. On sham-operated rats, the bile duct was mobilized but not ligated. Rats were maintained on a standard rat pellet diet and tap water *ad libitum*. Animal studies were approved by the Animal Experiment Committee of the University and conducted humanely, in accordance with the *Guide for the Care and Use of Laboratory Animals *(National Academic Press, USA, 1996). There were five groups of rats: (a) control rats receiving 0.7% CMC, (b) BDL rats receiving 0.7% CMC, (c) BDL rats receiving silymarin (50 mg/kg, mixed with 0.7% CMC), (d) BDL rats receiving Arm (3 mg/kg), and (e) BDL rats receiving Arm (10 mg/kg). Arm or silymarin was given by gavage twice daily for 3 weeks starting from the onset of BDL. Three weeks after bile duct ligation or sham operation, the rats were examined for the parameters listed below. On the day of measurement, venous blood was withdrawn from each rat under anesthesia, and thereafter the rat was sacrificed by KCl injection to remove the liver for homogenization and biochemical analysis.

### Biochemical analysis of plasma

Blood samples were collected (6 ml each from the femoral vein) and immediately centrifuged at 1300 *g *at 4°C, and plasma samples were kept at -80°C for liver and renal function tests. Alanine transaminase (ALT) and aspartate transaminase (AST) activities were measured using a colorimetric analyzer (Dri-Chem 3000, Fuji Photo Film Co, Tokyo, Japan), as we described previously [22].

### Histological examination

For morphometric studies, the liver fragments were taken from the left lobe of each rat. Liver specimens were preserved in 4% buffered paraformaldehyde and dehydrated in a graded alcohol series. Following xylene treatment, the specimens were embedded in paraffin blocks, cut into 5-μm thick sections and placed on glass slides. The sections were then stained with picro-sirius red for collagen distribution [27]. A numerical scoring system for histologically assessing the extent of fibrosis was adapted from the formula of Scheuer [28], with minor modifications [29]. Briefly, fibrosis was graded as: 0: no fibrosis; grade 1: enlarged, fibrous portal tracts; grade 2: periportal or portal-portal septa, but intact architecture; grade 3: fibrosis with architectural distortion; grade 4: probable or definite cirrhosis. Additionally, hepatocyte necrosis or degeneration severity was also graded as: 0, no hepatocyte necrosis or degeneration; grade 1, focal necrosis or degeneration of hepatocytes (mild, lesion no.≦ 3); grade 2, multifocal necrosis or degeneration of hepatocytes (moderate, lesion no.> 3); grade 3, locally extensive or diffuse necrosis or degeneration of hepatocytes (severe). Hepatocyte degeneration is mainly associated with cytoplasmic vacuolation and swelling, with the nuclear contour generally intact, whereas hepatocyte necrosis is associated with karyopyknosis (nuclear shrinkage) and karyorrhexis (nuclear rupture), in addition to degenerative changes. The liver scoring examination was performed by the pathologist (Y.-T. C.) who was blinded to rats' treatment assignment. Fibrosis scores were given after the pathologist had examined throughout three different areas in the tissue slide for each rat.

### Quantification of collagen deposition in the livers of BDL rats

A portion of liver tissue was homogenized in acetic acid (0.5 M) at 4°C using an ULTRA TURRAX^® ^homogenizer (Ika Labotechnik, Staufen, Germany). The fraction of insoluble collagen after acid extraction, composed of cross-linked collagen, was then heated at 80°C for 60 min for conversion into soluble gelatin. The gelatin contents of the acid extracts were assayed using the Sircol collagen assay kit according to the manufacturer's instructions and the method described previously [21,29].

### Quantitative real-time PCR for the analysis of gene transcripts

Total RNA was isolated from hepatic tissues by the method of Chomczynski and Sacchi [30]. For cDNA synthesis, 1 μg of total RNA was reverse-transcribed in a 30 μl of reaction mixture containing 10 μM dNTP mix, 500 μg/μl oligo(dT)_12-18_, 0.2 μM DTT, 40 units of RNase inhibitor, 200 units of M-MLV reverse transcriptase, and 5 × buffer (1.5 mM MgCl_2_) (Invitrogen, Carlsbad, CA, USA). The reaction mix was incubated at 37°C for 60 min and then denatured at 65°C for 10 min. Quantitative PCR analysis was performed using an ABI prism 7900 HT Sequence Detection System (Applied Biosystems, Foster City, CA, USA). In the study, we used two different methods of quantitative PCR [20-22]: (I) SyBR Green method for the expressions of *G3PDH, ICAM-1, TGF-β1*, and *α-SMA *genes. SyBR Green, a double-stranded DNA binding dye, was used for the fluorescent detection of DNA generated during the PCR. The PCR reaction was performed in a total volume of 20 μl with 0.4 pmol/μl of each primer, and 2 × SyBR Green PCR master mix (Applied Biosystems); 1 μl cDNA corresponding to 100 ng of total RNA was used as template. The primer sequences for PCR amplification (*α-SMA *forward primer: 5'-TTC GTT ACT ACT GCT GAG CGT GAG A-3', reverse primer: 5'-AAA GAT GGC TGG AAG AGG GTC-3'; *TGF-β1 *forward primer: 5'-TAT AGC AAC AAT TCC TGG CG-3', reverse primer: 5'-TGC TGT CAC AGG AGC AGTG-3'; *G3PDH *forward primer: 5'-AGC CCA GAA CAT CAT CCC TG-3', reverse primer: 5'-CAC CAC CTT CTT GAT GTC ATC-3') were according to our previous reports [20-22]. The primers of *ICAM-1 *(forward primer: 5'-CAC TAG AGG AGT GAG CAG GTT AAC AT-3', reverse primer: 5'-TAT GAC TCG TGA AAG AAA TCA GCT CTT-3') were designed according to the published sequence of rat ICAM-1 (D00913) for PCR amplification. (II) The Taqman^® ^PCR Core reagent kit (PE Applied Biosystems) was used according to the manufacturer's protocol for the expressions of *procollagen I (col 1α2), tissue inhibitor of metalloproteinase-1 (TIMP-1), iNOS, interleukin (IL)-6 *and *metallothionein *genes. Specific primers and probe for *procollagen I (col 1α2), tissue inhibitor of metalloproteinase-1 (TIMP-1), iNOS, interleukin (IL)-6 *and *metallothionein *genes were synthesized by PE Applied Biosystems. For each sample tested, PCR reaction was carried out in a 50-μl volume containing 1 μl of cDNA reaction (equivalent to 50 ng of template RNA) and 2.5 units of AmpliTaq Gold. Oligonucleotide primers and fluorogenic probe were added to a final concentration of 100 nM each. The amplification step consisted of 60 cycles of 94°C for 45 s, 58°C for 45 s, and 65°C for 1 min.

### Data analysis

Data are expressed as the mean ± SEM. One-way analysis of variance (ANOVA) was used for comparison of biochemical and molecular parameters. Statistical significance was accepted at *p *< 0.05. A non-parametric method (the Dunn procedure under the Kruskal-Wallis test) was used for multiple pairwise comparisons between groups for the histological grades of fibrosis. Statistical significance was accepted at *p *< 0.05.

## Results

### *In vitro *effects of Arm on HSC-T6 cells

#### Effects of Arm on TNF-α-induced collagen deposition in HSCs

**Figure 1:**
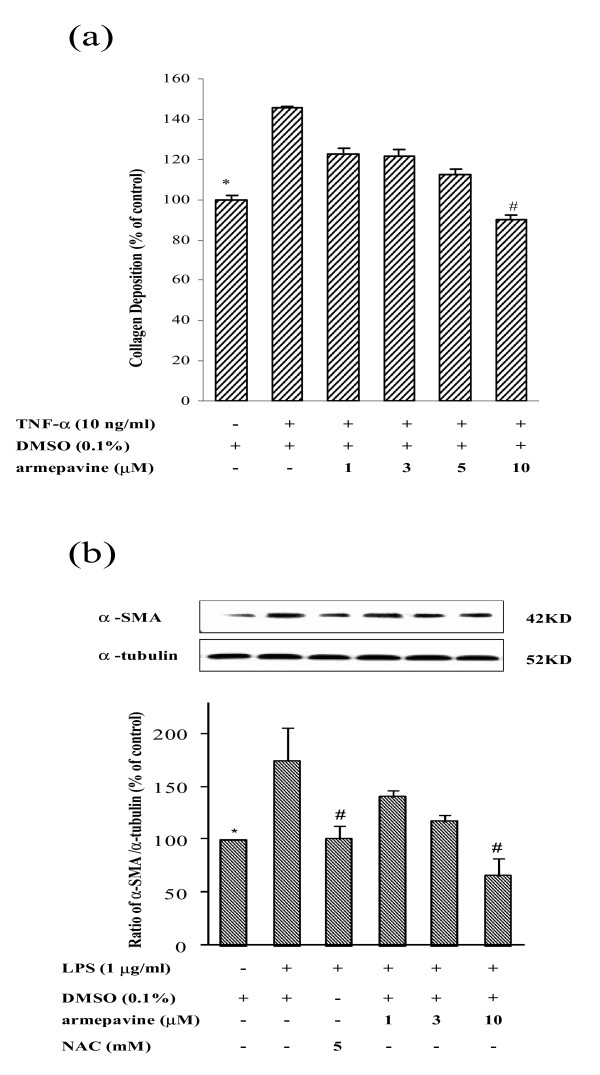
**(a) Effects of armepavine on collagen deposition by HSC-T6 cells after TNF-α stimulation for 24 hrs**. Collagen deposition by HSC-T6 cells was quantified by Sircol collagen assay (n = 3). *p < 0.05 for TNF-α alone *vs*. Control; ^#^*p *< 0.05 for TNF-α + armepavine *vs*. TNF-α alone. **(b) Armepavine reduced the protein expression of α-SMA induced by lipopolysaccharide (LPS, 1 μg/ml) in HSC-T6 cells for 24 hrs**. Representative results from three independent experiments are shown here, with *N*-acetylcysteine (NAC) as a positive control. *p < 0.05 for LPS alone *vs*. Control; ^#^*p *< 0.05 for LPS + armepavine or NAC *vs*. LPS alone.

As shown in Figure 1a, TNF-α (10 ng/ml) stimulated collagen deposition in HSC-T6 cells to 143 ± 9% of the un-stimulated, control level, which was concentration-dependently reduced by Arm (1 - 10 μM). Significant inhibition was observed at 10 μM (to 90 ± 2% of control level).

#### Effects of Arm on TNF-α- and LPS-induced α-SMA expression in HSCs

TNF-α (10 ng/ml) stimulated α-SMA secretion (to 274 ± 34% of control level) in HSC-T6 cells. Both *N*-acetylcysteine (NAC, 5 mM, a positive-control inhibitor) and Arm (1, 3 and 10 μM) reduced TNF-α-stimulated α-SMA secretion (to 101 ± 8% for NAC, 160 ± 4%, 127 ± 4% and 106 ± 8%, respectively, of control level) in HSCs.

LPS (1 μg/ml) also stimulated α-SMA secretion (to 174 ± 37% of control level) in HSC-T6 cells. Both NAC and Arm (1 - 10 μM) reduced LPS-stimulated α-SMA secretion in HSCs (Fig. 1b). Arm at the concentration range of 1-10 μM did not affect HSC cell viability in the MTT assay. Cell viability for Arm at 10 μM was 98 ± 4% of controls and not different from controls.

#### Effects of Arm on TNF-α-induced AP-1 and NFκB luciferase reporter gene assays in HSCs

**Figure 2:**
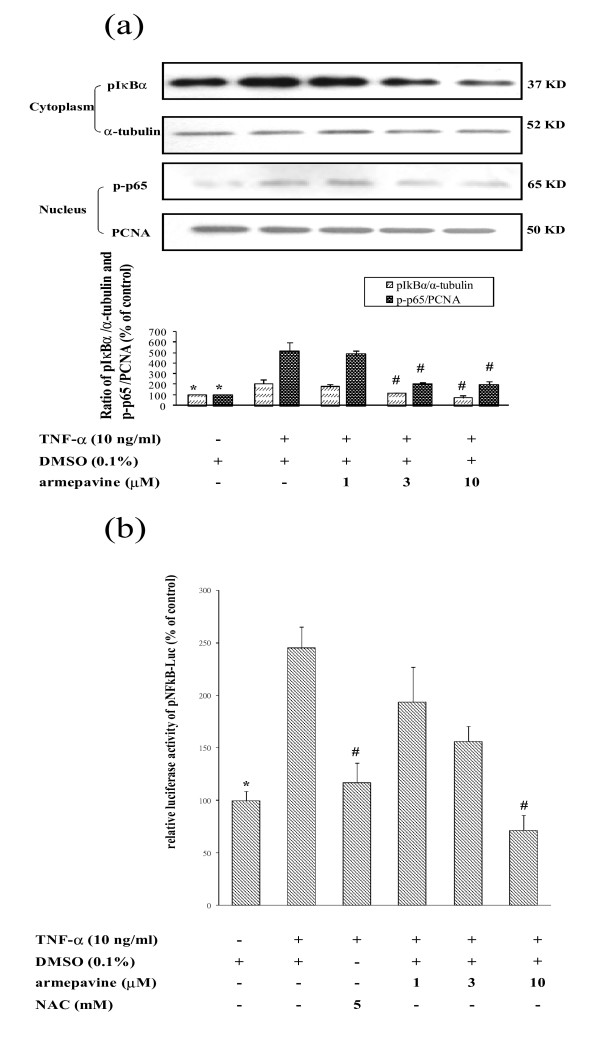
**(a) Effects of armepavine on TNF-α-induced IκBα phosphorylation in the cytoplasmic extract and translocation of NFκB in the nuclear extract of HSC-T6 cells for 6 hrs, with PCNA and α-tubulin as internal controls**. **(b) Effects of armepavine on TNF-α-induced NFκB transcriptional activity using luciferase reporter gene assay in HSC-T6 cells for 6 hrs, with *N*-acetylcysteine (NAC) as a positive control**. Representative results from three independent experiments are shown here *p < 0.05 for TNF-α alone *vs*. Control; ^#^*p *< 0.05 for TNF-α + armepavine or NAC *vs*. TNF-α alone.

HSC-T6 cells were transfected with pAP-1-Luc plasmid, which contains the AP-1-responsive region followed by the firefly *luciferase *gene. After exogenously adding luciferin to cell lysates, the luciferase-luciferin reactions generated luminescence with high sensitivity and could be measured. The luciferase levels of AP-1 in HSCs under TNF-α (10 ng/ml) were increased to 362 ± 36% of control level, which were concentration-dependently reduced by Arm (1, 3, and 10 μM, to 330 ± 49%, 274 ± 47% and 184 ± 13%, respectively, of control level, with significant difference at 10 μM). Arm (3 and 10 μM) also significantly attenuated LPS-stiumulated AP-1 luciferase levels (from 720 ± 43% to 360 ± 50% and 240 ± 115%, respectively, of control level) in HSCs. Similarly, TNF-α (10 ng/ml) significantly increased cytosolic IκBα phosphorylation and the NFκB (p65) protein amount in nuclear extracts of cells, indicative of nuclear translocation of active NFκB subunit. Both cytosolic IκBα phosphorylation and nuclear NFκB (p65) protein amount were concentration-dependently reduced by Arm (1 - 10 μM) (Fig. 2a). Moreover, Arm (1 - 10 μM) concentration-dependently reduced TNF-α-induced NFκB luciferase levels in HSCs (Fig. 2b).

#### Effects of Arm on TNF-α-stimulated MAPK phosphorylations

**Figure 3:**
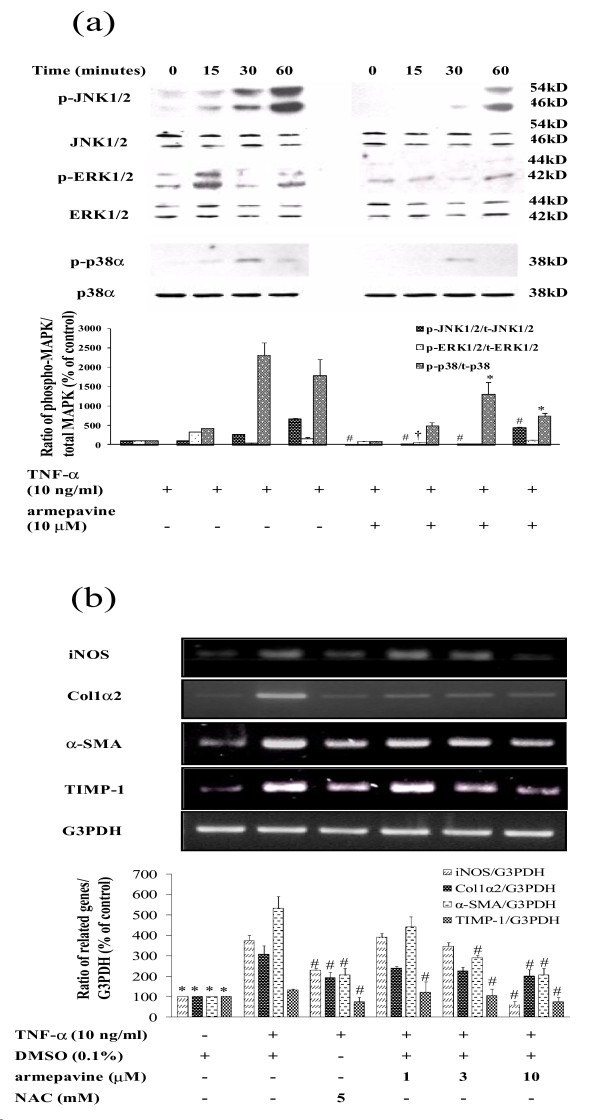
**(a) Effects of armepavine on TNF-α-induced ERK1/2, p38 and JNK 1/2 phosphorylation levels in HSC-T6 cells**. HSC-T6 cells were treated with TNF-α for 0-120 minutes. Representative results from three independent experiments are shown here. ^#^*p *< 0.05 for phosphorylation of JNK 1/2 induced by TNF-α + armepavine *vs*. TNF-α alone at the same time point; ^†^*p *< 0.05 for phosphorylation of ERK 1/2 induced by TNF-α + armepavine *vs*. TNF-α alone at the same time point; *p < 0.05 for phosphorylation of p38 induced by TNF-α + armepavine *vs*. TNF-α alone at the same time point. **(b) Quantitative real-time PCR analysis for the expressions of *iNOS, procollagen type I *(*Col 1α2*), *α-SMA *and *TIMP-1 *genes in HSC-T6 cells treated with TNF-α and armepavine for 24 hrs**. Representative results from three independent experiments are shown here, with *N*-acetylcysteine (NAC) as a positive control. *p < 0.05 for TNF-α alone *vs*. Control; ^#^*p *< 0.05 for TNF-α + (S)-armepavine or NAC *vs*. TNF-α alone.

The phosphorylations of MAP kinases are involved in HSC activation [1,4]. TNF-α-induced MAPK (p38, ERK1/2, and JNK) phosphorylations were analyzed by Western blot analyses of cell lysates of HSCs. As shown in Figure 3a, stimulation with TNF-α for up to 60 min significantly increased the levels of phosphorylated p38, ERK1/2, and JNK as compared to the unstimulated control. Peak ERK1/2, p38, and JNK phosphorylations occurred at 15, 30, and 60 min., respectively, and Arm (10 μM) attenuated all these phosphorylations.

#### Effects of Arm on mRNA expression levels of iNOS, collagen 1α2, TIMP-1 and α-SMA genes in HSCs

Figure 3b shows that TNF-α (10 ng/ml) stimulated mRNA expression levels of *iNOS, collagen 1α2, TIMP-1 and α-SMA *genes in HSC-T6 cells, as shown by quantitative real-time PCR analysis of gene transcripts. Arm (1 - 10 μM) concentration-dependently reduced TNF-α-induced transcriptions of these 4 fibrosis-related genes.

### *In vivo *effects of Arm on BDL rats

#### General features

The body weight of BDL rats was significantly lower than that of sham rats. BDL rats also displayed a sickened appearance, including less vigorous movements and less-smooth fir. The body weight of BDL rats was increased in the rats treated with either the low or high dose of Arm (Table 1). BDL rats showed an increase in liver weight (29.2 ± 0.4 *vs*. 19.5 ± 0.8 g, *p *< 0.05) compared with sham rats, a sign of hepatomegaly. Both Arm and silymarin treatment mildly decreased the liver weight in BDL rats (Table 1).

**Table 1:** General profiles in sham-operated (SO) and bile-duct-ligated (BDL) rats receiving armepavine (Arm), silymarin (sil) or vehicle (0.7% CMC) treatment.

**Group**	**SO +**	**BDL +**	**BDL + sil**	**BDL + Arm**	**BDL + Arm**
	**vehicle**	**vehicle**	**(50 mg/kg)**	**(3 mg/kg)**	**(10 mg/kg)**
BW (g)	386 ± 22	302 ± 21*	335 ± 17.8*^,#^	333 ± 16.1*^,#^	345 ± 22.0*^,#^
LW (g)	19.5 ± 0.8	29.2 ± 0.4*	27.4 ± 0.1*^,#^	27.5 ± 1.1*^,#^	26.3 ± 0.3*^,#^
ALT (U/L)	76 ± 5.0	756 ± 19*	545 ± 10*^,#^	536 ± 4 *^,#^	434 ± 11*^,#^
AST (U/L)	43 ± 2	168 ± 21*	128 ± 8*^,#^	127 ± 10*^,#^	73 ± 9*^,##^
Collagen (mg/g liver)	3.42 ± 0.59	6.63 ± 0.31*	5.36 ± 0.12*^,#^	6.18 ± 0.16*	4.38 ± 0.33*^,#^
α-SMA/α-tubulin ratio (%)	100 ± 1	182 ± 8*	150 ± 3*^,#^	173 ± 8*	141 ± 12*^,#^
Fibrosis score	0 ± 0	2.42 ± 0.20*	1.42 ± 0.20*^,#^	1.85 ± 0.14*^,#^	1.28 ± 0.18*^,##^
Necrosis score	0 ± 0	1.17 ± 0.31**	0.29 ± 0.18*^,##^	0.38 ± 0.18*^,#^	0.50 ± 0.22*^,#^

**Note**: BDL, bile-duct ligated; BW, body weight; LW, liver weight; ALT, alanine transaminase; AST, aspartate transaminase; collagen content (mg/g liver dry mass); protein expression of -SMA (expressed as -SMA/-tubulin ratio) in the cytoplasmic extract of liver tissues in Western blot analysis, with sham-operated group set as 100%; fibrosis and necrosis scores. Data are expressed as the mean ± SEM. The number of rats in each group is 6. *p < 0.05 vs. sham operated group; **p < 0.01 vs. sham operated group; ^#^p < 0.05 vs. BDL group; ^##^p < 0.01 vs. BDL group.

#### Plasma biochemistry

BDL rats showed significantly higher plasma ALT (756 ± 19 *vs*. 76 ± 5 U/ml, *p *< 0.01) and AST (168 ± 21 *vs*. 43 ± 2 U/ml, *p *< 0.01) levels compared with sham rats, indicating hepatic injury. Levels of both ALT and AST in BDL rats were significantly decreased by treatment of low-dose- or high-dose-Arm and silymarin, suggesting that Arm and silymarin ameliorated hepatic injury in BDL rats (Table 1).

#### Histological examination

**Figure 4:**
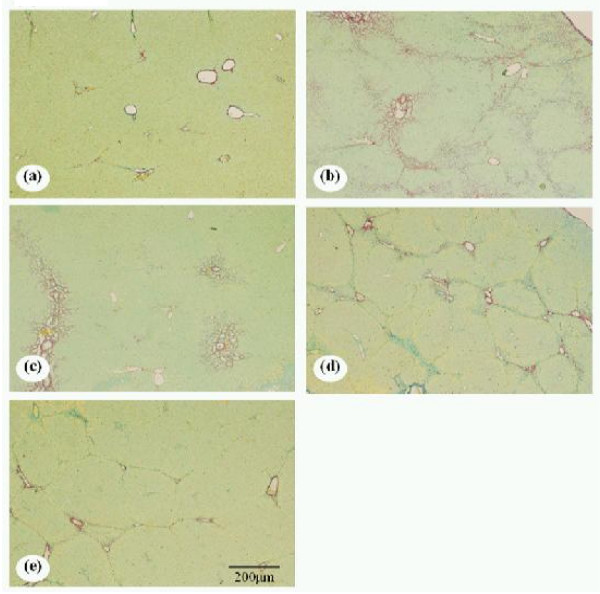
**Histological examination of liver sections in control and bile-duct-ligated (BDL) rats**. Representative liver sections were obtained from sham-operated rats **(a)**, BDL rats receiving vehicle **(b)**, BDL rats receiving 50 mg/kg silymarin **(c)**, BDL rats receiving 3-mg/kg armepavine **(d)**, and BDL rats receiving 10-mg/kg armepavine **(e)**. Sections were stained with Sirius red. Scale bar = 200 m.

Histological examination of livers from BDL rats revealed the following changes: progressive increase and expansion of fibrous septa and loss of hepatocytes, compared with control rats. Collagen fibers, as stained by Sirius-red, were distinctly deposited in the liver of BDL rats. Treatment with either Arm or silymarin decreased collagen deposition (Fig. 4). Confocal microscopy of double staining for α-SMA (red) and NFκB (green) also indicates that the α-SMA-positive cells with NFκB nuclear translocation were reduced with the high and low doses of Arm, and with silymarin (figures not shown). As shown in Table 1, BDL rats showed significantly higher fibrosis scores than sham rats. Fibrosis scores of livers from BDL rats were significantly reduced by treatment of Arm or silymarin (Table 1). The fibrosis scores were reduced by 47% and 24% with the high and low doses of Arm, respectively, and by 41% with silymarin. Hepatocyte necrosis scores in the livers from BDL rats were significantly reduced in rats treated with the high and low doses of Arm, and silymarin (Table 1), in line with amelioration of hepatic injury as indicated by plasma ALT and AST profiles. The hepatocyte necrosis scores were reduced by 57% and 68% with the high and low doses of Arm, respectively, and by 75% with silymarin. The possibility of adverse effects of high-dose Arm remains to be clarified in future studies.

#### Hepatic collagen content and α-SMA expression

Hepatic collagen levels were significantly increased in BDL rats compared with control rats (6.63 ± 0.31 *vs*. 3.42 ± 0.59 mg/g liver weight, *p *< 0.01), suggesting abundant accumulation of collagen in the liver of BDL rats. Hepatic collagen levels were significantly decreased by high-dose Arm (4.38 ± 0.33 mg/g, *p *< 0.01), and silymarin (5.36 ± 0.12 mg/g, *p *< 0.05), suggesting that Arm and silymarin ameliorated hepatic collagen deposition in BDL rats. α-SMA protein expression was increased significantly in the liver tissues of BDL rats compared with control rats, as detected by Western blot analysis. Treatment with high-dose Arm or silymarin reduced α-SMA protein expression significantly (Table 1).

*Analysis of transcripts of procollagen I (col 1α2), transforming growth factor-β 1 (TGF-β1)*, *tissue inhibitor of metalloproteinase-1 (TIMP-1), intercellular adhesion molecule-I (ICAM-1)*, *iNOS, interleukin (IL)-6 *and *metallothionein genes*

There were significant increases in hepatic mRNA expressions of *procollagen I (col 1α2), transforming growth factor-β1 (TGF-β1)*, *tissue inhibitor of metalloproteinase-1 (TIMP-1), intercellular adhesion molecule-I (ICAM-1)*, *iNOS*, and *interleukin (IL)-6 *genes relative to *G3PDH *in BDL rats compared with control rats (Table 2). The mRNA expression levels of profibrogenic genes in BDL rats were all attenuated in Arm and silymarin-treated groups, suggesting that fibrosis-related gene transcripts were attenuated by Arm or silymarin treatment. On the other hand, the hepatic mRNA expression of *metallothionein *gene in BDL rats was significantly reduced compared with control rats. High-dose Arm and silymarin treatment significantly increased the hepatic mRNA expression of *metallothionein *gene in BDL rats (Table 2).

**Table 2:** Quantitative real-time PCR analysis for the mRNA expressions in sham-operated (SO) and bile-duct-ligated (BDL) rats receiving armepavine (Arm), silymarin (sil) or vehicle (0.7% CMC) treatment

**Group**	**SO +**	**BDL +**	**BDL + sil**	**BDL + Arm**	**BDL + Arm**
	**vehicle**	**vehicle**	**(50 mg/kg)**	**(3 mg/kg)**	**(10 mg/kg)**
*col1α2/GAPDH*	1.00 ± 0.44	12.5 ± 0.2*	7.31 ± 0.11*^,#^	7.45 ± 0.08*^,#^	5.09 ± 0.23*^,#^
*TGF-β1/GAPDH*	1.00 ± 0.81	21.5 ± 0.2*	13.1 ± 0.9*^,#^	14.5 ± 0.8*^,#^	7.09 ± 1.72*^,#^
*TIMP-1/GAPDH*	1.01 ± 0.84	28.0 ± 2.6*	4.93 ± 1.88*^,#^	4.36 ± 1.70*^,#^	3.87 ± 1.59*^,#^
*ICAM-1/GAPDH*	1.00 ± 0.20	22.5 ± 0.5*	12.2 ± 0.1*^,#^	15.5 ± 0.2*^,#^	7.03 ± 0.35*^,#^
*iNOS/GAPDH*	1.00 ± 0.08	7.53 ± 0.17*	5.31 ± 0.09*^,#^	6.45 ± 0.08*^,#^	3.09 ± 0.31*^,#^
*IL-6/GAPDH*	1.00 ± 0.10	31.5 ± 0.3*	11.3 ± 0.1*^,#^	10.5 ± 0.1*^,#^	4.09 ± 0.17*^,#^
*Metallothionein/GAPDH*	1.00 ± 0.10	0.34 ± 0.06*	0.56 ± 0.08*^,#^	0.37 ± 0.10*	0.57 ± 0.05*^,#^

**Note**: Quantitative real-time PCR analysis for the expressions of *procollagen I (col 12), transforming growth factor-β1 (TGF-β1)*, *tissue inhibitor of metalloproteinase-1 (TIMP-1), intercellular adhension molecule-I (ICAM-1)*, *iNOS, interleukin (IL)-6 *and *metallothionein *genes in sham-operated rats and BDL rats receiving saline, Arm (3 and 10 mg/kg) or silymarin (50 mg/kg). Data are expressed as the mean ± SEM. The number of rats in each group is 6. *p < 0.05 vs. sham operated group; ^#^*p *< 0.05 vs. BDL group.

## Discussion

In the present study, we observed *in vitro *that Arm exerted in HSC-T6 cells several inhibitory effects, including (a) attenuation of TNF-α-induced collagen deposition and α-SMA protein expression, (b) inhibition of TNF-α-induced NFκB and AP-1 activities, together with IκBα phosphorylation and NFκB p65 nuclear translocation, (c) down-regulation of mRNA expressions of *iNOS, collagen 1α2, TIMP-1 and α-SMA *genes, (d) attenuation of LPS-induced α-SMA protein expression and AP-1 activities, (e) suppression of TNF-α-induced MAPK (p38, ERK1/2, and JNK) phosphorylations. Our *in vivo *study showed Arm exerted inhibitory effects on hepatic fibrosis in BDL rats, including (a) reduction of hepatic fibrosis scores and collagen contents of livers in BDL rats, (b) attenuation of hepatic injury in terms of plasma ALT and AST levels, (c) reduction of hepatic mRNA expression levels of *procollagen I (col 1α2), transforming growth factor-β1 (TGF-β1), tissue inhibitor of metalloproteinase-1 (TIMP-1), intercellular adhesion molecule-I (ICAM-1), iNOS*, and *interleukin (IL)-6 *genes, and (d) improvement of hepatic mRNA expression level of *metallothionein *gene. Overall, there was dose-dependent *in vivo *therapeuctic effects of Arm in the (a), (b), (c), and (d) parameters. To our knowledge, the present study was the first to demonstrate both *in vitro *inhibitory effects of Arm on TNF-α-induced NFκB and AP-1 activities as well as collagen deposition in a cell line of rat hepatic stellate cells, and *in vivo *anti-fibrotic effects of Arm on hepatic fibrosis in BDL rats.

Activation of NFκB signaling pathways is well documented to result in enhanced transcription of both *iNOS *and *ICAM-1 *genes [6-8]. We propose that Arm exerted inhibitory effects primarily on NFκB signaling pathways in HSCs, and thereby led to its *in vitro *and *in vivo *anti-fibrogenic effects. In the Arm-treated BDL rats, the number of α-SMA-positive cells was significantly decreased. Overall, treatments with Arm (10 mg/kg) yielded better therapeutic benefits than silymarin (50 mg/kg) in terms of reductions in plasma AST and ALT activities, hepatic collagen contents, and fibrosis-related mRNA expressions of *TGF-β1, collagen Iα2, iNOS, ICAM-1*, and *IL-6 *genes. Improvement of hepatic mRNA expression level of *metallothionein *gene was also better achieved in BDL rats receiving Arm than silymarin treatment.

In our *in vitro *studies, we used *N*-acetylcysteine (NAC) for comparison with Arm as NAC has been known to inhibit TNFα-induced NFκB activity in HSCs [22] and T lymphocytes [31]. Silymarin, an extract from milk thistle (*Silybum marianum*) was included in the *in vivo *study for comparison with Arm, as it has a long history of usage by patients with liver diseases [32,33] and has been reported in the literature to exert anti-fibrotic effects in bile-duct-obstructed and DMN-intoxicated rats [20,34,35]. The dosage of silymarin (50 mg/kg) was chosen as our previous studies have demonstrated its *in vivo *anti-fibrotic effects [20-22].

BDL has been used to produce a reliable experimental model due to high yield of liver fibrosis [36]. In our previous studies, we have observed that oral administration of tetrandrine (1 and 5 mg/kg, bid) for 3 weeks, an alkaloid isolated from Chinese medicinal herb *Stephania tetrandrine *into BDL rats could also ameliorate hepatic fibrosis [20]. Although Arm doses (3 and 10 mg/kg, bid) used in the present study were higher than tetrandrine doses, the percentage reduction in fibrosis scores was also higher in Arm than tetrandrine (24% vs. 14% for low-dose regimen and 47% vs. 38% for high-dose regime, respectively) [20].

Metallothionein is reported to control intracellular redox status and regulate the activity of NFκB and other redox-regulated transcription factors [37]. In the literature, it has been reported that transfection of the *metallothionein *gene inhibits TNF-α-induced IκB degradation and suppresses NFκB-dependent gene expression induced by TNF-α [37]. Moreover, *metallothionein *gene therapy has been reported to attenuate hepatic fibrosis induced by carbon tetrachloride (CCl_4_) in mice [38]. We also found that addition of TNF-α to HSC-T6 cells resulted in the reduction of *metallothionein *gene expression (data not shown). In our previous study, we have shown that hepatic mRNA expression of *metallothionein was *down-regulated in BDL rats compared to sham rats, suggesting potential impairment of an intracellular mechanism against oxidative stress [20]. In the present study, we found that down-regulation of hepatic *metallothionein *mRNA expression in BDL rats was partially corrected by high-dose Arm or silymarin treatment. Taken together, *in vivo *anti-fibrotic effects of Arm and silymarin were associated with improved *metallothionein *mRNA expression in BDL rats.

Mitogen-activated protein kinases (MAPKs) are proposed to play a key role in intracellular signaling cascades in normal and pathogenetic conditions [39,40]. ERK1/2, JNK, and p38 are the three major members of MAPKs and reported to be associated with cellular oxidative stress, inflammation, proliferation and migration [39,40]. There are also cross-talks between MAPK and NFκB signaling pathways in cellular oxidative stress and inflammation [41]. In our *in vitro *study, we observed that both MAPK and NFκB signaling pathways were activated by TNF-α, which were attenuated by Arm in HSCs. It remains to be delineated how Arm inhibited TNF-α-induced MAPK and NFκB signaling cascades, with respect to cross-talks or networks and the diverse molecules involved in HSCs.

Although there are as yet no clinically efficacious anti-fibrotic agents, experimental studies have been unremittingly conducted to assess the potentials of agents targeting the reduction of inflammation, inhibition of HSC activation or proliferation, induction of HSC apoptosis, or promotion of scar matrix degradation [1-5]. Recently, there are some interesting reports of plant-derived anti-fibrotic agents in experimental animals [32,42,43]. Herbs or their active principles such as Sho-saiko-to [42,44,45], silymarin [34,35], Inchin-ko-to [46], *Salvia miltiorrhiza *[29,47], curcumin [48] and tetrandrine [20,22], *etc*., have been shown respectively to reduce the severity of hepatic fibrosis in treated rats. These experimental studies suggest the potential of anti-fibrotic agents from herbs.

In summary, our study results showed that Arm exerted both *in vitro *and *in vivo *antifibrotic effects in rats, possibly through anti-NF-κB activation pathways. And it remains to be confirmed for its therapeutic benefits in other animal models (such as carbon tetrachloride or dimethylnitrosamine intoxication) of hepatic fibrosis before proposing its validation of clinical efficacy using randomized controlled trials [1-5].

## Abbreviations

AP-1: activating protein-1; α-SMA: α-smooth muscle actin; ERK: extracellular signal-regulated kinase; G3PDH: glyceraldehyde-3-phosphate dehydrogenase; HSC: hepatic stellate cell; ICAM-1: intercellular adhension molecule 1; IL-6: interleukin-6; iNOS: inducible nitric oxide synthase; JNK: c-jun N-terminal kinase; MAPKs: mitogen-activated protein kinases; NFκB: nuclear factor-κB; TNF-α: tumor necrosis factor-α; TGF-β1: transforming growth factor-β1; TIMP-1: tissue inhibitor of metalloproteinase-1.

## Competing interests

The authors declare that they have no competing interests.

## Authors' contributions

T-CW carried out *in vitro *and *in vivo *bioassays and drafted the manuscript. C-CS participated in the design of the study and synthesized Arm. Y-TC performed histopathological and immunohistochemical examinations. C-DK and Y-LL participated in the design and coordination of the study. Y-TH conceived of the study, and participated in its design and coordination and helped to draft the manuscript. All authors read and approved the final manuscript.
